# Impact of the COVID-19 pandemic on the mental health of frontline healthcare workers in a highly affected region in Brazil

**DOI:** 10.1186/s12888-023-04702-2

**Published:** 2023-04-17

**Authors:** Mírian Cohen, Luciane Nascimento Cruz, Ricardo Bertoglio Cardoso, Maria de Fátima Pessoa Militão de Albuquerque, Ulisses Ramos Montarroyos, Wayner Vieira de Souza, Ana Bernarda Ludermir, Maria Rosimery de Carvalho, Julianne Damiana da Silva Vicente, Marcelo Paulino Viegas Filho, Fanny Julia Mireille Cortes, Marina Teixeira de Siqueira Silva, Carla Menezes Cavalcante Almeida, Luana Nepomuceno Gondim Costa Lima, Maria Amelia de Sousa Mascena Veras, Carl Kendall, Ligia Regina Franco Sansigolo Kerr, Celina Maria Turchi Martelli, Suzi Alves Camey

**Affiliations:** 1 Graduate Studies Program in Epidemiology, Federal University of Rio Grande do Sul (UFRGS), Ramiro Barcelos St, 2400, 2nd fl, Porto Alegre, Rio Grande do Sul 90035-003 Brazil; 2 National Institute of Science and Technology for Health Technology Assessment (IATS), Porto Alegre, Rio Grande do Sul Brazil; 3 Hospital Moinhos de Vento (HMV), Porto Alegre, Rio Grande do Sul Brazil; 4 Institute Aggeu Magalhães, FIOCRUZ-PE, Recife, Pernambuco Brazil; 5 University of Pernambuco, Recife, Pernambuco Brazil; 6 Federal University of Pernambuco, Recife, Pernambuco Brazil; 7 University of Pará State, Belém, Pará Brazil; 8 Faculty of Medical Sciences, Santa Casa de São Paulo, São Paulo, Brazil; 9 Department of Community Health, Federal University of Ceará, Fortaleza, Ceará Brazil; 10 Tulane University School of Public Health and Tropical Medicine, New Orleans, LA USA; 11 Statistics Department, Federal University of Rio Grande do Sul (UFRGS), Porto Alegre, Rio Grande do Sul Brazil; 12 Hospital de Clínicas de Porto Alegre (HCPA), Porto Alegre, Rio Grande do Sul Brazil

**Keywords:** COVID-19, Mental health, Healthcare workers, Anxiety, Depression, PTSD.

## Abstract

**Background:**

The COVID-19 pandemic had a major impact on the mental health of healthcare workers (HCWs), especially in low and middle-income countries, which had to face additional political, social, and economic challenges. We thus aimed to assess the prevalence of mental health outcomes and the associated factors in HCWs treating COVID-19 patients in one of the most affected regions in Brazil.

**Methods:**

We used the Respondent-Driven Sampling method to assess the risks of COVID-19 infection and symptoms of mental disorders in nurses, nursing technicians, and physicians who worked on the frontline in the metropolitan region of Recife. 865 healthcare workers completed a survey regarding sociodemographic data, work-related risks, and symptoms of mental disorders - SRQ-20 for common mental disorders (CMD); AUDIT-C for problematic alcohol use; GAD-7 for anxiety; PHQ-9 for depression; PCL-5 for post-traumatic stress disorder (PTSD). Gile’s successive sampling estimator was used to produce the weighted estimates by professional category. A Poisson regression model with robust variance was used to analyze factors associated with a positive screening for CMD. We will present the results of a cross-sectional analysis of the mental health outcomes after the first peak of COVID-19 – from August 2020 to February 2021.

**Results:**

The prevalence ratios for a positive screening for CMD were 34.9% (95% CI: 27.8–41.9) in nurses, 28.6% (95% CI: 21.3–36.0) in physicians, and 26.6% (95% CI: 16.8–36.5) in nursing technicians. Nurses presented a higher prevalence of depressive symptoms (23%). Positive screening for problematic alcohol use (10.5 to14.0%), anxiety (10.4 to 13.3%), and PTSD (3.3 to 4.4%) were similar between the professional categories. The main factors associated with CMD in nurses and physicians were related to an intrinsic susceptibility to mental illness, such as previous or family history of psychiatric disorder, and female sex. Among nurse technicians, work-related factors, such as accidents with biological material, presented the strongest association with CMD.

**Conclusion:**

The mental health of HCWs fighting COVID-19 in Recife was severely affected. It is crucial that healthcare services provide adequate working conditions and psychological support, investing in programs to promote and protect HCWs mental health.

## Introduction

The global COVID-19 health crisis began as an outbreak of pneumonia caused by the SARS-COV-2 virus in late 2019 in the city of Wuhan, China. Due to its rapid spread, in March 2020 the World Health Organization (WHO) defined COVID-19 as a pandemic [1]. The first measures recommended to prevent transmission of the infection were identifying and isolating suspected cases, quarantining for contacts, social distancing, and hygiene measures (hand washing and respiratory etiquette) [2]. With a high potential for morbidity and mortality, the absence of a specific treatment, and the lack of vaccines, a sense of panic arose worldwide.

In Brazil, the spread of the disease was exacerbated by political, economic, and ideological factors. Brazil has been one of the most heavily impacted countries by the pandemic, with high numbers of cases and deaths [3, 4]. Several factors have contributed to this situation, such as the high vulnerability of a large part of the population, high levels of informal work, no centralized national policy to combat the pandemic, the fragility of the epidemiological surveillance system, a lack of testing, and a lack of clear, unified communication based on scientific evidence about best practices recommended for the population. The northeast region of the country, considered one of the poorest, was also one of the most heavily affected [3–6].

Although the pandemic reached every segment of the population, it is understood that healthcare workers (HCWs), who were exposed to the continuous risk of infection and work overload, have been affected differently, especially those working on the front lines assisting COVID-19 patients at the beginning of the pandemic [7, 8]. These professionals have had to face several challenges, such as the fear of infection and contaminating their families, lack of experience treating the disease, lack of personal protective equipment (PPE), insufficiency of tests, stigmatization, work overload, and the collapse of the health system [8, 9]. This scenario has led to emotional suffering, psychological stress, and burnout, which in some cases worsened, leading to psychiatric disorders [10].

The first studies about the impact of the COVID-19 pandemic on the mental health of Chinese HCWs showed high rates of symptoms of emotional distress, depression, anxiety, and insomnia [9, 10]. In a recent review, Aymerich [11] showed that this situation occurred in several countries. Similar studies have been conducted in Brazil [12, 13], finding a high prevalence of psychiatric symptoms, such as stress (55.8%), depression (57.2%), and anxiety (46.20%) [12]. However, Brazil’s large territory covers very heterogeneous regions with different social, economic, infrastructure, and health system conditions. In addition, the different regions of the country faced different epidemic moments over time [13].

Brazil has five geographic regions – North, Northeast, Central-West, Southeast, and South. The Northeast and North regions have the worst socioeconomic indicators. Significant social vulnerabilities and health inequalities have aggravated the COVID-19 crisis in these localities. The state of Pernambuco, in the Northeast region, reached one of the highest mortality rates from COVID-19 in Brazil in 2020 [6]. The literature is still scarce regarding the impact of the pandemic on the mental health of HCWs who were most exposed to stressful situations in Brazil, i.e., those working on the front line in one of the most affected regions of the country.

Hence, the objective of this study was to assess the prevalence of mental health outcomes among frontline HCWs in the metropolitan region of Recife, capital of Pernambuco. We also sought to identify factors associated with symptoms of common mental disorders (CMD) according to the professional category. Our hypothesis is that the factors associated with CMD differ among HCWs categories.

## Methodology

### Participants and procedures

A longitudinal study entitled “*Fique Seguro* (Stay Safe)” was carried out using the respondent-driven sampling (RDS) method to assess the risks of COVID-19 infection and mental health outcomes in healthcare professionals working on the front line caring for COVID-19 patients in the metropolitan region of Recife, Pernambuco, northeastern Brazil [14]. The metropolitan region of Recife includes 15 municipalities, with a total population of approximately 4 million residents, which is 42% of the state’s population [15]. This article will present the results of a cross-sectional analysis of baseline mental health outcomes data collected between August 2020 and February 2021.

HCWs were eligible to participate in the study if they met the following inclusion criteria: (a) professional category of physicians, nurses, and nurse technicians; (b) working on the frontline (defined as attending suspected or confirmed patients with COVID-19); c) working on the metropolitan region of Recife; d) accepted the consent form. The participants could be working in hospitals, clinics, or primary care services. As exclusion criteria, the HCWs could not be away from their professional activity.

We chose to use the RDS online method due to (1) mobility difficulties during the pandemic due to lockdowns and other strict distancing measures, (2) to protect the field team, and (3) the difficulty of accessing frontline healthcare workers because they had to take sick leave if infected (minimum of 15 days) or were relocated to work in other sectors.

A formative survey was conducted through in-depth interviews using a quick online ethnographic assessment of a convenience sample of physicians, nurses, and nurse technicians. Approximately 9 frontline HCWs per category were interviewed. The objective was to evaluate the best strategy for recruiting participants and to identify facilitating factors and potential difficulties. Since the pandemic led to work overload in this group, it was necessary to adapt traditional RDS recruiting. Thus, after the healthcare workers recommended other potential participants, the research team contacted them directly. To facilitate contact, we asked the participants to advise their colleagues beforehand that the research team would be contacting them. Initially, five seeds were selected for each professional category, who then recommended another five possible participants, and so on. No financial incentive was offered for participation or recruiting colleagues. We sent thank you messages to the participants, reinforcing the relevance of their participation in the study, which was helping call attention to the pandemic’s impact on the physical and mental health of HCWs.

Professionals were invited to participate in the study through WhatsApp messages or phone calls. If they accepted, a WhatsApp link was provided that directed them to an online App containing the consent form and questionnaire. After they signed the consent form, a questionnaire could be answered on electronic devices such as cell phones, tablets, or computers. More details about the methodology can be found in an article by Albuquerque [14]. This study followed the STROBE-RDS guidelines [16].

### Variables and outcomes

The following variables were included in the questionnaire:

1. Sex, age, and self-reported clinical comorbidities (such as diabetes mellitus, hypertension, cardiopathy, nephropathy, obesity, and others);
2. Professional category (physician, nurse, nurse technician), number of places that work, work setting (intensive care unit [ICU], emergency, outpatient, inpatient clinics, others), work sector (public, private, both), number of days assisting patients with COVID-19, frequency of N95 use (always, not always), training on PPE use (yes, no), accidents with biological material (yes, no);
3. Risk perception (none, low, medium, high), confidence level of avoiding infection (none, some, confident, completely confident), work overload due to the pandemic (yes, no), family isolation due to the pandemic (yes, no);
4. COVID-19 infection, work leave, and hospitalization;
5. Self-reported previous history of psychiatric diagnosis (yes, no), current psychiatric treatment (yes, no), family history of psychiatric diagnosis (yes, no), work leave due to a mental health condition (yes, no).

The question about risk perception was “How do you characterize the risk of being infected by Covid-19”, and about the confidence level of avoiding infection was “How confident are you that you can avoid being infected?”. The variable frequency of N95 use was considered “always” if the HCW used the N95 mask more than 95% of the time when providing care to patients with suspected COVID-19. COVID-19 infection was considered “yes” if the HCW had tested positive on polymerase chain reaction (PCR) or rapid immunoglobulin tests. The risk questions in the questionnaire were based on the WHO Guide for Risk Assessment of Health Professionals, developed as an interim guidance at the beginning of the pandemic [17].

To assess mental health outcomes, we used standardized, self-administered, easy-to-understand screening questionnaires that had been translated and validated for the Brazilian population [18–22]. The screening was performed for symptoms of depression, anxiety, post-traumatic stress disorder (PTSD), and problems related to alcohol abuse. The outcomes were measured using the following instruments:

The *Self-Report Questionnaire (SRQ-20)* assesses symptoms of common mental disorders (CMD). It consists of 20 “yes/no” questions. Individuals with at least 8 “yes” responses were considered positive [18]. The *shortened version of the Alcohol Use Disorders Identification Test (AUDIT-C)* was used to screen for problematic alcohol use. It includes 3 questions that are scored from 0 to 4. The final score is obtained by summing the answers to the 3 questions, with total scores ranging from 0 to 12. A minimum score of 6 points was considered positive [19]. Participants with positive results on either of these scales, suggesting the presence of symptoms of mental disorders, were asked to respond to questionnaires assessing symptoms of specific mental disorders, which are described below.

The *Patient Health Questionnaire (PHQ-9)*, which is used to identify depressive symptoms, consists of 9 questions that assess each symptom of a major depressive episode according to the Diagnostic and Statistical Manual of Mental Disorders, 4th Edition, Text Revision (DSM-IV). The frequency of each symptom over the past two weeks is scored on a Likert scale from 0 (none) to 3 (almost every day). The final score is the sum of the scores of the 9 questions. Scores ≥ 9 were considered positive [22].

The *Generalized Anxiety Disorder 7-item scale (GAD-7)* screens for symptoms of anxiety disorders. Its 7 items ask about the frequency of anxiety symptoms in the last 2 weeks, which are evaluated using a Likert scale scored from 0 (never) to 3 (almost every day). The total score is the sum of the response values. Scores ≥ 10 were considered positive [20].

The *Post-traumatic Stress Disorder Checklist for DSM-5 (PCL-5)* screens for symptoms of Post-Traumatic Stress Disorder (PTSD) according to Diagnostic and Statistical Manual of Mental Disorders, 5th Edition (DSM-V) criteria. Its 20 items are answered on a Likert scale from 0 (not at all) to 4 (extremely), with total scores being the sum of the responses. Scores ≥ 36 were considered positive [21].

### Statistical analysis

Given the difficulty of using conventional sampling without estimates of the outcomes of interest, we defined a sample size of 350 healthcare workers in each category. This number had been used in several previous RDS surveys and mandated by the Ministry of Health and consultants. It has been our experience that convergence (or arriving at the same or similar value in chains from different seeds) on main variables is achieved with this sample size, as it proved to be here. The samples discussed in Gile [23] also achieved convergence long before 350.

To estimate the size of the participant’s social network, we use the question “*How many colleagues are closest to you that would you invite to participate in this study?*” To avoid social network sizes that would severely affect the weights of individuals, we assigned a value of 3 to the participant’s social network if the reported number was a value less than 3, and a maximum of 150 if the reported number was greater than this value. We also measured homophily in each professional category. Homophily estimates the tendency of participants to recruit individuals with similar characteristics to their own [24].

We used Gile’s successive sampling estimator to produce category-weighted estimates. We considered that each HCW category had distinct personal social networks, as they differ in terms of socioeconomic, work activities, working conditions, PPE access, and use, risk, effect on homelife and other topics. Since this estimator assumes a finite population and requires an estimate of the size of this population for each group, we used the population of physicians registered as active in the Pernambuco Regional Councils of Medicine and nurses and nurse technicians in the Regional Councils of Nursing.

Categorical variables were presented as proportions and 95% confidence intervals (95% CI); continuous variables were presented as mean and 95% CI. Prevalence ratios (PR) for the CMD outcome were estimated using a Poisson regression model with robust variance. First, we performed bivariate analyses to investigate the association between the studied variables and the prevalence of positive screening for CMD. After, variables that presented p < 0.20 in the bivariate analyses were included in a multivariate model in a single block without exclusions. We also tested for multicollinearity, variables were excluded when VIF > 5. Estimates and analyses were performed using the RDS and the survey package of R software version 4.0.3 [25–28].

### Ethical issues

This project was approved by the National Research Ethics Committee (CONEP; CAAE: 30629220.8.0000.0008) and the Ethics and Research Committee of the Hospital Moinhos de Vento (CAAE: 33653220.0.1001.5330). Providing electronic informed consent was mandatory to participate and access the questionnaire. Participants gave informed consent to participate in the study before taking part. This study was performed in accordance with the ethical standards laid down in the 1964 Declaration of Helsinki and its later amendments.

Participants who were positive on any of the screening scales received a warning message, suggesting they should consult a healthcare professional for further symptom assessment.

## Results

### RDS and sociodemographic variables

A total of 865 frontline HCWs were interviewed, including 374 nurses, 307 physicians, and 185 nurse technicians. The mean network size was 8 for nurses and physicians and 12 for nurse technicians. Ten waves were reached in the nurses’ category, 13 in the physicians’ category, and 14 in the technicians’ category. The homophily related to the positive screening for CMD was 1.0 in nurses, 0.97 in physicians, and 0.97 in nurse technicians.

The sample was mostly women; the lowest percentage of women was among physicians (62.0%; 95% CI: 54.0-69.9). The median participant age was similar between categories – among 30 and 40 years old. Regarding race, the lowest proportion of non-whites was among physicians (26.7%; 95% CI: 18.5–34.8) and the highest proportion was among nurse technicians (68.3%; 95% CI: 58.3–78.3). However, a considerable portion of the participants in all categories did not report their race (Table 1).

**Table 1:** Percentage (95% CI) of the sample characteristics according to the professional category

	Nurses	Nurse technicians	Physicians
	n = 374	n = 185	n = 307
**Female Sex**	86.6 (81.3–91.9)	86.8 (80.7–93.0)	62.0 (54.0-69.9)
Age (years)*	36 (29; 42)	37 (29; 44)	29 (26; 33)
**Race**			
White	31.4 (24.6–38.3)	18.7 (10.9–26.4)	34.9 (27.0-42.8)
Non-White	50.6 (43.2–58.0)	68.3 (58.3–78.3)	26.7 (18.5–34.8)
Missing	18.1 (12.6–23.8)	13.0 (5.5–20.5)	38.4 (30.6–46.2)
**Comorbidities**	31.2 (24.4–38.1)	29.3 (18.9–39.8)	21.1 (14.5–27.8)
**Workplace > 1**	59.4 (51.9–66.7)	64.7 (53.1–76.3)	90.2 (85.0-95.4)
**Work Setting**			
Emergency/ICU	61.0 (53.6–68.4)	74.8 (63.0-86.6)	88.2 (82.9–93.5)
Other	39.0 (31.7–46.5)	25.2 (13.4–37.1)	11.8 (6.5–17.1)
**Work Sector**			
Public	78.8 (72.1–85.5)	77.4 (66.4–88.5)	43.3 (35.3–48.9)
Private	8.9 (3.8–14.0)	8.0 (0-17.9)	5.4 (1.4–9.4)
Both	12.3 (7.1–17.4)	14.6 (7.1–22.1)	51.3 (43.1–59.6)
Time assisting patients with COVID-19 (days)*	178 (120; 200)	153 (90; 185)	150 (120; 180)
**Frequency of N95 use**			
Always	57.7 (50.4–65.0)	69.7 (58.1–81.2)	58.4 (50.3–66.6)
Not always	36.1 (29.1–43.2)	26.9 (15.9–37.8)	40.1 (32.0-48.3)
Missing	6.2 (2.6–9.8)	3.5 (0-9.3)	1.4 (0.1–2.8)
**Training on PPE use**	72.4 (66.7–79.1)	83.3 (73.7–92.8)	74.0 (67.5–80.6)
**Accident with biological material**	11.5 (6.7–16.3)	13.1 (6.4–19.9)	15.1 (10.4–19.9)
**Risk Perception**			
None/Low	6.4 (2.2–10.7)	5.3 (0-11.1)	4.7 (0.7–8.7)
Medium/High	93.6 (89.3–97.8)	94.7 (88.9–100.0)	95.3 (91.3–99.3)
**Confidence level of avoiding infection**			
None/Some	48.6 (41.2–55.9)	48.7 (37.2–60.1)	56.2 (48.0-64.5)
Confident/Completely Confident	51.4 (44.1–58.8)	51.3 (40.0-62.8)	43.8 (35.6–52.0)
**Work overload due to pandemic**	83.9 (79.2–88.5)	69.5 (57.6–81.5)	87.3 (82.5–92.1)
**Family isolation due to pandemic**	66.0 (59.2–72.7)	64.3 (52.9–75.6)	70.5 (63.3–77.8)
**COVID-19 infection**	29.7 (23.2–36.3)	30.1 (19.9–40.3)	37.4 (29.6–45.2)
**Work leave due to suspected COVID-19 infection**	45.3 (37.9–52.7)	37.1 (25.8–48.4)	48.5 (40.2–56.7)
**Hospitalization due to COVID-19** ** infection**	1.3 (0.7-2.0)	5.5 (0-11.6)	2.3 (0-5.5)
**Previous history of psychiatric diagnosis**	16.6 (11.5–21.7)	7.2 (2.9–11.4)	24.6 (17.2–32.1)
**Current psychiatric treatment**	13.4 (8.8–18.1)	2.3 (0-4.8)	16.2 (10.4–22.0)
**Family history of psychiatric disorders**	33.2 (26.2–40.3)	28.6 (18.9–38.4)	48.8 (40.6–57.1)
**Work leave due to mental health condition**	5.2 (1.1–9.2)	3.9 (0-9.9)	3.6 (1.3–5.8)

ICU = Intensive Care Unit; PPE = Personal Protective Equipment

*median (IQR)

### Work-related and risk perception variables

Most participants worked in emergency and intensive care units and reported work overload due to the pandemic. More than half of the participants in each category reported always using an N95 mask at work and having received training in the use of personal protective equipment. The proportion of individuals who mentioned accidents with biological material at work was similar in all three categories, ranging from 11.5 to 15.1%. The risk of contamination in the workplace was considered medium or high by the vast majority of the participants; more than half reported having isolated themselves from their families due to the pandemic. The rate of COVID-19 infection ranged from 29.7% (95% CI: 23.2–36.3) in nurses to 37.4% (95% CI: 29.6–45.2) in physicians. These professionals had worked on average for 5 months caring for COVID-19 patients.

### Mental health outcomes

Approximately 34.9% (95% CI: 27.8–41.9) of the nurses, 28.6% (95% CI: 21.3–36.0) of the physicians, and 26.6% (95% CI: 16.8–36.5) of the nurse technicians showed symptoms of CMD. Regarding problematic alcohol use, 10.5–14.0% of these professionals screened positive.

More nurses took work leaves due to mental health distress (5.2%; 95% CI: 1.1–9.2). Nursing technicians were the group that had the lowest percentage of current psychiatric treatment (2.3%; 95% CI: 0-4.8). Table 2 shows the prevalence of CMD, problematic alcohol use, depression, anxiety, and PTSD symptoms according to the professional category.

**Table 2:** Prevalence of positive screening (95% CI) in mental health outcomes according to the professional category

	Nurses	Nurse technicians	Physicians
**SRQ-20**			
Prevalence	34.9 (27.8–41.9)	26.6 (16.8–36.5)	28.6 (21.3–36.0)
**AUDIT-C**			
Prevalence	10.5 (6.4–14.5)	13.6 (6.7–20.4)	14.0 (7.9–20.2)
**PHQ-9**			
Prevalence	22.3 (15.7–28.9)	15.7 (6.9–24.5)	18.3 (12.2–24.5)
**GAD-7**			
Prevalence	13.3 (8.5–18.1)	10.4 (2.8–18.1)	15.2 (9.5–20.9)
**PCL-5**			
Prevalence	3.2 (0.6–5.8)	3.7 (0-7.5)	4.4 (0-9.4)

SRQ-20 = Self-Report Questionnaire; AUDIT-C = shortened version of the Alcohol Use Disorders Identification Test; PHQ-9 = Patient Health Questionnaire; GAD-7 = Generalized Anxiety Disorder 7-item; PCL-5 = Post-traumatic Stress Disorder Checklist for DSM-5

The results of the regression analyses for each professional category are shown in Table 3. In the multivariate analysis for nurses, the only factor that remained associated with positive screening for CMD was previous history of psychiatric disorders (PR = 2, 05; 95% CI: 1.39–3.01). The only associated factors for nurse technicians were accidents with biological material (PR = 2.53; 95% CI: 1.66–3.85) and work leave due to mental health distress (PR = 3.03; 95% CI: 1.68–5.47). The factors associated with an increase in the prevalence of positive screening for CMD in physicians were female sex (PR = 2.29; 95% CI: 1.38–3.81), previous history of mental disorders (PR = 1.50 (95% CI: 1.06-2.12), and family history of mental disorders (PR = 2.09; 95% CI: 1.27–3.43).

**Table 3:** Crude and adjusted prevalence ratios (95% CI) for the Common Mental Disorder outcome

	Nurses		Nurse technicians		Physicians	
	Crude PR (95% CI)	Adjusted PR (95% CI)	Crude PR (95% CI)	Adjusted PR (95% CI)	Crude PR (95% CI)	Adjusted PR (95% CI)
**Sex**						
Male	1.00	1.00	1.00		1.00	1.00
Female	1.54 (0.89–2.67)	1.40 (0.84–2.35)	1.45 (0.54–3.94)		2.34 (1.41–3.89)	2.29 (1.38–3.81)
**Age**	0.98 (0.96-1.00)		0.99 (0.96–1.02)		1.00 (0.96–1.03)	
**Race**						
Non-White	1.00	1.00	1.00		1.00	
White	1.03 (0.72–1.49)	1.06 (0.74–1.52)	1.09 (0.42–2.83)		1.33 (0.87–2.03)	
Missing	1.34 (0.85–2.11)	1.32 (0.91–1.92)	1.80 (0.69–4.84)		1.55 (0.93–2.59)	
**Comorbidities**						
No	1.00	1.00	1.00	1.00	1.00	
Yes	1.27 (0.91–1.78)	1.25 (0.91–1.73)	1.64 (0.88–3.07)	1.46 (0.94–2.26)	1.51 (1.01–2.27)	
**Workplace > 1**						
No	1.00	1.00	1.00		1.00	
Yes	1.27 (0.89–1.82)	1.28 (0.93–1.77)	0.83 (0.34–2.01)		1.26 (0.61–2.61)	
**Work Setting**						
Emergency/ICU	1.00		1.00	1.00	1.00	
Other	0.84 (0.56–1.28)		0.96 (0.30–3.08)	0.83 (0.37–1.90)	0.85 (0.40–1.80)	
**Work Sector**						
Both	1.00		1.00		1.00	
Private	0.96 (0.47–1.94)		2.10 (0.81–5.44)		1.36 (0.61–3.02)	
Public	0.67 (0.44–1.04)		1.45 (0.60–3.48)		1.08 (0.68–1.71)	
**Time assisting COVID-19 patients (days)**	1.000 (0.998–1.003)		1.000 (0.996–1.004)		1.000 (0.996–1.004)	
**Frequency of N95 use**						
Always	1.00	1.00	1.00		1.00	
Not always	1.21 (0.85–1.72)	1.14 (0.84–1.56)	1.20 (0.57–2.53)		1.16 (0.76–1.79)	
**Training on PPE use**						
No	1.00		1.00		1.00	1.00
Yes	0.82 (0.55–1.22)		1.26 (0.62–2.56)		0.98 (0.63–1.52)	0.94 (0.65–1.34)
**Accident with biological material**						
No	1.00		1.00	1.00	1.00	
Yes	1.05 (0.62–1.79)		2.68 (1.55–4.61)	2.53 (1.66–3.85)	1.17 (0.68–2.02)	
**Risk perception**						
Medium/High	1.00		1.00	1.00	1.00	
None/Low	0.96 (0.46-2.00)		0.35 (0.06–3.08)	0.37 (0.05–2.45)	0.59 (0.22–1.58)	
**Confidence level about avoiding infection**						
Confident/Completely Confident	1.00	1.00	1.00	1.00	1.00	
None/Some	1.16 (0.81–1.67)	1.21 (0.86–1.71)	2.27 (1.01–5.11)	1.92 (0.97–3.79)	0.87 (0.58–1.30)	
**Work overload due to pandemic**						
No	1.00	1.00	1.00		1.00	
Yes	1.31 (0.66–2.59)	1.45 (0.77–2.74)	1.10 (0.47–2.57)		0.80 (0.37–1.71)	
**Family isolation due to pandemic**						
No	1.00		1.00	1.00	1.00	
Yes	1.10 (0.77–1.57)		1.51 (0.74–3.10)	1.84 (1.00-3.38)	1.00 (0.68–1.48)	
**COVID-19 infection**						
No	1.00	1.00	1.00		1.00	1.00
Yes	1.26 (0.89–1.79)	1.30 (0.94–1.80)	1.11 (0.60–2.06)		0.69 (0.39–1.24)	0.72 (0.46–1.15)
**Work leave due to suspected COVID-19 infection**						
No	1.00		1.00	1.00	1.00	
Yes	1.13 (0.81–1.57)		0.98 (0.51–1.88)	1.09 (0.65–1.84)	0.92 (0.60–1.42)	
**Hospitalization due to COVID-19**						
No	1.00	1.00	1.00		1.00	
Yes	0.20 (0.04–0.91)	0.21 (0.04–1.08)	0.99 (0.28–3.51)		1.35 (0.57–3.23)	
**Previous history of psychiatric diagnosis**						
No	1.00	1.00	1.00	1.00	1.00	1.00
Yes	2.02 (1.40–2.92)	2.05 (1.39–3.01)	1.33 (0.46–3.85)	1.16 (0.50–2.73)	1.35 (0.94–1.94)	1.50 (1.06–2.12)
**Family history of psychiatric disorder**						
No	1.00	1.00	1.00	1.00	1.00	1.00
Yes	1.14 (0.79–1.64)	1.06 (0.75–1.50)	1.30 (0.68–2.49)	1.22 (0.71–2.11)	2.00 (1.18–3.40)	2.09 (1.27–3.43)
**Mental health leave**						
No	1.00	1.00	1.00	1.00	1.00	
Yes	1.27 (0.74–2.19)	1.09 (0.64–1.86)	2.60 (0.98–6.90)	2.94 (1.49–5.77)	1.16 (0.55–2.42)	

PR = Prevalence Ratio; Adjusted PR: the models were adjusted for variables with p < 0.2 in the bivariate model

# the race variable was not included in the adjusted analysis due to multicollinearity.

ICU =  IntensiveCare Unit; PPE = Personal Protective Equipment.

## Discussion

To our knowledge, this is the largest study with frontline healthcare workers on this topic that has been conducted in Brazil. Our study showed that frontline HCWs in the metropolitan region of Recife had a high prevalence of symptoms indicative of common mental disorders, such as depression and anxiety. These results confirm the findings of other international studies regarding the negative impact of the COVID-19 pandemic on the mental health of these professionals [9–11, 29, 30]. Our sample consisted mostly of female healthcare workers aged approximately 30 years, which has also been found in other studies, given that most health professionals are women and that older workers are put on leave due to the risk of complications once infected with COVID-19 [13, 31, 32]. A minority of participants reported their race as White, drawing attention that more than half of the physicians and approximately one-third of nurses and nurse technicians did not report their race.

Although a lack of masks and other personal protective equipment was common at the beginning of the pandemic, in our study slightly more than half of the participants reported always using an N95 mask at work. However, this number is still low, considering the severity of the disease. This finding could be related to the fact that more than half of the respondents worked in emergency or intensive care units, which were prioritized in the distribution of these materials, in addition to already using this equipment in everyday work routines. A previous study in Recife (Albuquerque 2021) reinforced the gravity of the situation, showing that 49.3% of physicians and 28.6% of nurses who worked on the front lines reported using the same N95 mask for more than eight days, which could indicate that the availability reported in our study may have been due more to reuse than adequate availability of masks.

It is interesting to note that of all 3 groups, nursing technicians were the category that least reported having a previous diagnosis of mental health and undergoing psychiatric treatment. In our understanding, this is probably due to underdiagnosis, since their socioeconomic status is lower than physicians and nurses [33], making access to evaluation and specialized medical treatment more difficult. The smaller sample of nurse technicians may also have been due to similar reasons. These professionals usually have a heavier workload when compared to doctors and nurses, so perhaps they had less time available to answer the survey. To complete the online questionnaire, quality internet was required, and one possible explanation is that some of these professionals may not have had adequate cell phones and sufficient data plans. Furthermore, physicians and nurses may have a better understanding of how research methodology works, being more interested in participating. Regarding the prevalence of COVID-19 infection, although the rates were similar among the 3 categories, hospitalization rates were higher among nurse technicians, reflecting greater vulnerability, including a higher prevalence of comorbidities, which could also be attributed to socioeconomic disparities.

In our study, the high prevalence of CMD symptoms was similar among physicians, nurses, and nurse technicians. In 2021 Kunz [34] conducted a review on the pandemic’s psychological impact on physicians and nurses, finding worse mental health outcomes among nurses. Even considering the higher presence of the female gender in the profession, factors such as longer contact time and exposure to the emotional burden with patients were considered crucial.

It is interesting that the prevalence of PTSD symptoms in our sample was low, especially since several other studies have found rates 5 to 8 times higher among these workers [13, 29, 35]. One hypothesis is that professionals affected by more severe CMD symptoms would be unwilling to answer surveys at a time of extreme stress or might have been on leave due to mental health distress, being ineligible for the study. The point of the pandemic at which the data were collected, just after the first peak and before the second peak, must also be considered. PTSD symptoms may have already improved prior to the interview. In this sense, we also consider that although we found a high prevalence of CMD symptoms in our sample, we believe the actual prevalence may have been even higher.

It is important to highlight that we evaluated aspects such as previous and family history of psychiatric disorders, social isolation, hospitalization for COVID-19, and accidents with biological material in this study, variables that are extremely relevant to mental health outcomes and still receive little attention in most studies. In these 3 professional categories, we observed different factors associated with positive screening for CMD, reflecting a different behavior of these variables in each category. Factors that we expected to be associated with CMD symptoms, such as clinical comorbidities [36–38], work overload [7, 35, 39], lack of personal protective equipment [7, 37], concerns about infection [35, 36], and a personal history of COVID-19 infection [36, 40] were not confirmed in our sample.

Female sex, which several studies have found to be associated with symptoms of mental disorders [7, 40, 41], was only significant in our study among physicians. Among nurses, previous history of mental disorders was the only variable associated with CMD symptoms, whereas among physicians, the associated variables were previous and family history of mental disorders and female sex. These results could indicate that CMD symptoms in these professionals may be more related to the previous susceptibility to mental illness than to specific workplace experiences during the pandemic. Among nurse technicians, very different aspects were related to CMD symptoms, such as accidents with biological materials and mental health leaves. Compared to nurses and physicians, the nursing technician category seems to have more factors directly related to the pandemic associated with CMD symptoms.

Although some Brazilian studies have evaluated the mental health of health professionals during the pandemic [12, 13, 35], they have not focused on frontline HCWs. These workers tend to have higher rates of symptoms of mental disorders such as anxiety, insomnia, depression, and PTSD than general health professionals [29, 32]. In addition, the state of Pernambuco had the second highest COVID-19 fatality rate (5.32%) nationwide in 2020, almost twice that of the national rate (2.89%) [6], which probably impacted differently on the mental health of the professionals working in this region. However, it is not possible to generalize these results to other regions of the country, as the Northeast region of Brazil was one of the most impacted regions by the COVID-19 pandemic. Moreover, Brazil is a large country that presents important cultural, political, social, economic, and health system infrastructure differences throughout its territory. More studies are needed to assess mental health outcomes of HCWs in other regions of the country.

This study has certain limitations that should be reported. First, the RDS methodology was used in this population not because it is a traditional “hard to reach” population, but because it has become hard to reach as a result of the pandemic. Only among nurses was it possible to reach the pre-established sample size. This may have been due to the high demand for work of these professionals and the higher socioeconomic level of most of them. Previous Brazilian studies using RDS in other populations have found that since the social networks of individuals in better socioeconomic conditions tend to behave differently, RDS might not be the best way to access them [42]. However, the homophily values ​​found in the 3 groups were close to 1, indicating randomness regarding the CMD outcome. Second, we must have caution when interpreting positive screening results for mental health outcomes. These questionnaires do not provide a psychiatric diagnosis, which must be confirmed by a specialist. Third, it should also be noted that we could not establish causal relationships between the studied variables and the positive screening for CMD due to the cross-sectional design.

Our results showed a high prevalence of symptoms of common mental disorders in HCWs working in the frontline in the metropolitan region of Recife, Brazil. The main factors associated with CMD differed among professional categories. In nurses and physicians, these factors were more related to an intrinsic susceptibility to mental illness, and in nurse technicians, to workplace experiences during the pandemic. Our study demonstrated that it is crucial that healthcare services and the government pay attention to this situation, investing in the promotion and protection of the mental health of these professionals. Institutions must provide adequate and safe working conditions, as these are modifiable factors that can protect both the physical and mental health of HCWs. In Brazil, through an initiative with academic institutions, the Ministry of Health created a program providing free online psychological and psychiatric care for health professionals with emotional distress. Additionally, we also consider it extremely necessary that the institutions in which these professionals work develop psychological support programs and perform screening for psychiatric disorders with referral to treatment when indicated. We must remember that HCWs are fundamental for combating serious health situations such as the pandemic, and they must be valued and receive adequate attention to maintain their physical and mental integrity.

## Data Availability

Data and materials are available upon reasonable request. Proposals for the dataset (deidentified participant data, data dictionary) should be directed to the corresponding author: mirian.cohen@22c.com.br. To gain access, data requestors will need to present their plan of analysis and sign a data access agreement.
